# The zebrafish *prospero *homolog *prox1 *is required for mechanosensory hair cell differentiation and functionality in the lateral line

**DOI:** 10.1186/1471-213X-9-58

**Published:** 2009-11-30

**Authors:** Anna Pistocchi, Carmen G Feijóo, Pablo Cabrera, Eduardo J Villablanca, Miguel L Allende, Franco Cotelli

**Affiliations:** 1 Department of Biology, Università degli Studi di Milano, Via Celoria 26, 20133, Milan, Italy; 2 Center for Genomics of the Cell, Facultad de Ciencias, Universidad de Chile, Casilla 653, Santiago, Chile; 3 Departamento de Ciencias Biologicas, Facultad de Ciencias Biologicas, Universidad Andres Bello, Santiago, Chile; 4 International PhD Program in Molecular Medicine, Università "Vita-Salute" S Raffaele, Milan, Italy

## Abstract

**Background:**

The lateral line system in zebrafish is composed of a series of organs called neuromasts, which are distributed over the body surface. Neuromasts contain clusters of hair cells, surrounded by accessory cells.

**Results:**

In this report we describe zebrafish *prox1 *mRNA expression in the migrating primordium and in the neuromasts of the posterior lateral line. Furthermore, using an antibody against Prox1 we characterize expression of the protein in different cell types within neuromasts, and we show distribution among the supporting cells and hair cells.

**Conclusion:**

Functional analysis using antisense morpholinos indicates that *prox1 *activity is crucial for the hair cells to differentiate properly and acquire functionality, while having no role in development of other cell types in neuromasts.

## Background

The lateral line of fish and amphibians comprises a set of sensory organs, the neuromasts, arranged on the head and body surface in a species-specific pattern [1,2]. Within each neuromast there is a centrally located cluster of mechanosensory cells, the hair cells, which are functionally and morphologically equivalent to the mechanosensory hair cells of the vertebrate inner ear [3]. The hair cells are surrounded by a group of accessory cells of at least two types: mantle cells and supporting cells [4,5]. The hair cells can be evidenced easily in live fish because they incorporate fluorescent styryl dyes [6,7] or by labeling with anti-acetylated tubulin antibody [8].

The posterior lateral line (PLL) in the zebrafish larva consists of a single line of neuromasts running along the horizontal myoseptum of the trunk and tail; the neuromasts are innervated by afferences from the PLL ganglion located behind the otic vesicle. The neuromasts are deposited by the migration of a posterior lateral line placodal primordium (PLLP), from 20 until 42 hours post fertilization (hpf) [9]. By 72 hpf the pattern of neuromasts is complete: five to six neuromasts along each side of the body plus an additional cluster of two to three neuromasts at the end of the tail.

The *prox1 *homeobox gene is the vertebrate homolog of *prospero *in *Drosophila melanogaster *that is responsible for neuronal/glial fate of sibling cells during *Drosophila *embryonic development [10,11]. Prospero/Prox1 protein can act as transcriptional activator or repressor, depending on the target gene and subcellular distribution [12-14]. The protein structure is highly conserved in insects and vertebrates and contains both a nuclear localization signal (NLS) and a nuclear export signal (NES), regulated by a Prospero domain [15,16]. Several studies demonstrated that Prospero/Prox1 subcellular distribution can be either cytoplasmatic or nuclear, depending on the cell fate [11,15,16]. In fact, there is a direct correlation between Prox1, cell cycle regulation and cell fate specification during the development of several vertebrate organs such as the inner ear [17], liver [18], lens [19], lymphatic system [20,21], gustatory system [22], and central nervous system [23-25]. In the chick inner ear, Prox1 labels dividing progenitor supporting cells that are fated to become hair cells [26]. Thus, it is of interest to determine whether this gene is also expressed in the mechanosensory cells of the fish lateral line system.

Here, using *in situ *hybridization techniques in zebrafish embryos and larvae, we demonstrate that *prox1 *mRNA is expressed only in the PLLP and recently deposited neuromasts. Furthermore, we characterize Prox1 protein expression in 48 and 96 hpf fish using immunohistochemistry with an anti-Prox1 antibody in combination with other markers or transgenic lines expressing GFP in the diverse cell types of the PLL. Finally, we investigate the functional role of *prox1 *in PLL development by means of morpholino- and mRNA- microinjection to achieve loss- and gain-of-function, respectively. We show that *prox1 *does not participate in development of accessory cell types in the lateral line system, nor is it involved in the first stages of hair cell specification. However, we provide evidence that loss of *prox1 *function results in defects in hair cell differentiation, suggesting that it is a critical transcription factor for sensory function.

## Results and discussion

### *prox1 *expression in the lateral line primordium and neuromasts

**Figure 1:**
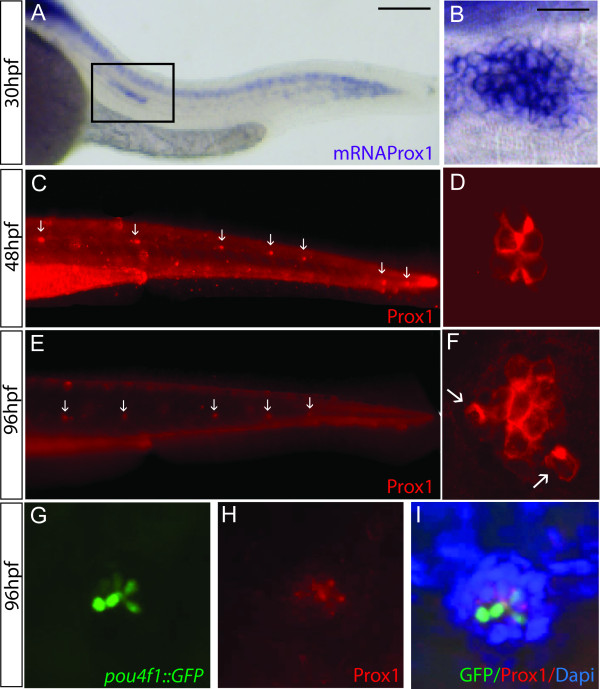
**Prox1 expression in the lateral line system of zebrafish embryos**. (A) *In situ *hybridization of *prox1 *at 30 hpf shows expression in the CNS and in the lateral line migrating primordium (box). (B) Enlarged view of a *prox1 *positive deposited neuromast in the posterior lateral line at 30 hpf. (C, E) Immunofluorescence using an anti-Prox1 antibody at 48 hpf and 96 hpf, arrows indicate the deposited neuromasts. (D, F) Close up of Prox1 expression in a neuromast at the two stages examined. (G, H, I) Immunofluorescence labeling in neuromasts with anti GFP (G), anti Prox1 (H) and the cell nuclei with DAPI (I) in 96 hpf *pou4f1::GFP *transgenic larvae. Scale bar = 10 micron.

A previous description of the *prox1 *mRNA expression pattern in zebrafish revealed that the gene is expressed, among other tissues, in the PLL system [25]. In zebrafish, the embryonic PLLP begins its migration at 20 hpf and reaches the tip of the tail at about 42 hpf. *prox1 *mRNA is detected during the entire journey of the migrating PLLP and shortly after deposition of the neuromasts (Fig. 1A and data not shown). Proneuromasts (neuromasts in which hair cells are yet to differentiate) also express *prox1 *mRNA, specifically in a group of cells at the center of the cell cluster, including the location where the hair cells will eventually arise (Fig. 1B). *prox1 *mRNA was not detected after 30 hpf, indicating a strong reduction in mRNA levels beginning at this time point.

To more precisely analyze the expression of the *prox1 *product in the lateral line system, we used an antibody against Prox1 [27] to carry out immunohistochemistry in zebrafish embryos and larvae. Prox1 protein expression had been described in cavefish lateral line hair cells [22] and in the lateral line primordium in zebrafish [28]. As previously shown by Roy and collegues [29], our initial immunostaining experiments confirmed that expression of Prox1 is detected extensively in muscle cells (not shown), which prevented us from clearly distinguishing the label in the overlying lateral line. Thus, in order to visualize expression in neuromasts, we used reduced amounts of detergent during immunolabeling to preclude penetrance of the antibody; in this fashion, we were able to obtain specific staining of superficially located cells (such as neuromast cells) without labeling the muscle cells (Fig. 1C and 1E). Prox1 expression was detected in few cells in each deposited neuromasts at 48 hpf (Fig. 1C-D) and 96 hpf (Fig. 1E-F), with the number of labeled cells increasing at the later developmental timepoint. At 48 hpf, immunolabel is seen in a small group of centrally located cells (4-8 cells) suggesting that expression occurs predominantly in mechanosensory hair cells and/or their precursors (Fig. 1D). At 96 hpf, the cluster of labeled cells is larger (6-12 cells) and we often observed labeling in more peripheral cells (arrows in Fig. 1F). Since the number of hair cells at this timepotint is, on average, around 10-12 [30], expression of Prox1 is likely to occur predominantly in hair cells. To confirm expression of Prox1 in hair cells, we perfomed immunostains against Prox1 in *pou4f1::GFP *transgenic larvae. This transgenic line carries a DNA construct that directs cytoplasmatic Green Fluorescent Protein (GFP) to hair cells, at different stages of their differentiation process [31]. Comparison of immunostaining (red label) and GFP expression (green label) at 96 hpf shows that Prox1 positive cells coincide, for the most part, with GFP-labeled cells (Fig. 1G-I). Most peripheral cells of the neuromast (labeled with DAPI in Fig. 1I) do not show staining. Prox1 label is seen in mature hair cells (strong GFP expressing cells in the center of the cluster) as well as in immature hair cells (weak GFP labeled cells). We conclude that Prox1 is predominantly expressed in cells that are committed to the hair cell lineage and in differentiating hair cells.

Our results show that *prox1 *mRNA is expressed at high levels during development of the lateral line system, but then diminishes as the system matures. Despite this reduction in mRNA expression, we observe strong protein label when using the anti-Prox1 antibody after neuromast deposition and in a group of centrally located cells as the neuromast matures. Therefore, high levels of protein expression follow a temporally distinct pattern to mRNA expression and could indicate that *prox1 *mRNA is short lasting while the protein is stable, at least in hair cells. More work will be required to determine whether this is indeed the case.

**Figure 2:**
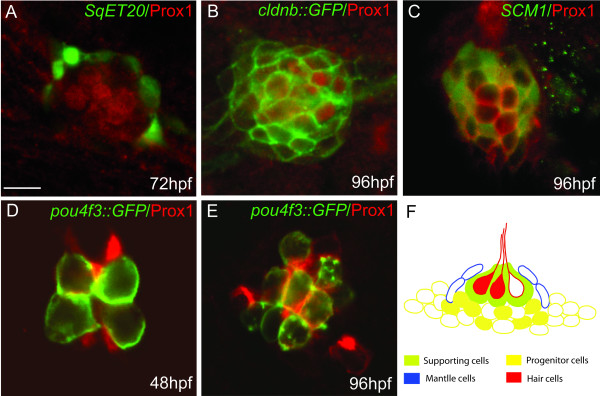
**Co-localization of Prox1 protein with different cell type markers in the lateral line system**. Immunofluorescence showing Prox1 (red) in different GFP transgenic lines (green) at 48, 72 and 96 hpf: (A) *SqET20 *that labels mantle cells; (B) *cldnb::GFP *that labels all cells that form the neuromast; (C) *SCM1 *that labels presumptive progenitor cells; (D, E) *pou4f3::GFP *that labels hair cells. (F) Schematic representation of a neuromast showing the different cell types. Prox1 expression is represented as filled cells and can be seen among hair cells (red) and underlying supporting cells (green) and/or progenitor cells (yellow). Prox1 is absent from mantle cells (blue). Scale bar = 5 micron.

To more accurately localize Prox1 protein expression to specific neuromast cells, we carried out immunostaining using additional transgenic zebrafish lines, which express GFP in the different cell types in the lateral line system (Fig. 2). The *SqET20 *transgenic line [32] labels the mantle cells in neuromasts, which surround the hair cells and provide a central opening for protrusion of kinocilia into the environment [30,33]. Visualization of both GFP and anti-Prox1 label (Fig. 2A) shows that Prox1-positive cells are contained within the ring of mantle cells, with little or no overlap between them (10 neuromasts analyzed). Therefore, Prox1 is likely to be expressed in hair cells and possibly in underlying progenitors and/or in supporting cells, but not in mantle cells. We next used two transgenic lines that label accessory cells in neuromasts: the *claudinB::GFP *line [34] that labels all accessory cells (Fig. 2B) and the *SCM1 *line [35] that labels all supporting cells (Fig. 2C). In these larvae, localization of the Prox1 signal in a subset of the GFP-labeled cells is observed but, clearly, not all supporting cells express Prox1. To determine the extent of overlap between Prox1 protein expression and lateral line hair cells, we used the *pou4f3::mGFP *line [36], in which GFP is directed to the membrane of differentiated hair cells (Fig. 2D-E). In the developmental stages analyzed (48 and 96 hpf) we observed a partial coincidence between the expression of both markers, indicating that Prox1 protein is present in some, but not all, differentiated hair cells and is also found in other (GFP negative) cells. These findings suggests that Prox1 may be expressed in a specific progenitor cell population or during early stages of maturation of hair cells, prior to the appearance of differentiation markers. This is consistent with the situation in the chick embryonic otocyst where cProx1 protein levels remain elevated in dividing sensory progenitor cells and in newly formed hair cells and supporting cells, and expression becomes down-regulated as these cells mature [26].

Our interpretation of the expression pattern of Prox1 protein is that it is likely to be expressed in a group of precursor cells, supporting cells, and in differentiating hair cells (Fig. 2F). After differentiation, Prox1 becomes down-regulated as it is not observed in all mature hair cells (Fig. 2E). Whether Prox1 expression is a marker for immediate hair cell progenitors that are fated to become hair cells [37], as occurs in the chick inner ear [26], will require further analysis. Studies performed in other species have demonstrated that Prox1 promotes terminal mitoses. For example in the ganglion mother cell (GMC) of *Drosophila*, the *prox1 *homolog *Prospero*, represses positive regulators of the cell cycle and diminishes mitotic activity [38,39]. Moreover, in the lens of *Prox1 *null mice, cells fail to correctly exit the cell cycle because of the delayed expression of negative regulators such as p27^kip1^, and their differentiation is altered [19].

### *prox1 *loss- and gain-of-function experiments

We sought to learn whether *prox1 *is important for PLL development in the zebrafish. We prevented translation of the gene by injecting, into one-cell stage embryos, 8 ng of a specific ATG-targeted antisense morpholino oligonucleotide (*prox1 *MO) that has been previously described [25,40,41]. The efficacy of the morpholino was tested with Prox1 immunostain experiments that show reduction of the protein levels in morphants compared to contro injected fish [see Additional file 1]. Identical results were obtained by injecting the ATG-targeted morpholino and a splice site morpholino, splice *prox1 *MO, indicating that the effect is specific to *prox1 *loss of function (data not shown). Control fish were injected with 8 ng of a non-specific morpholino which did not elicit a phenotype. As an additional functional assay, we microinjected *prox1 *mRNA in the same fashion to determine whether a gain-of-function experiment would be indicative of the role of this gene in zebrafish.

**Figure 3:**
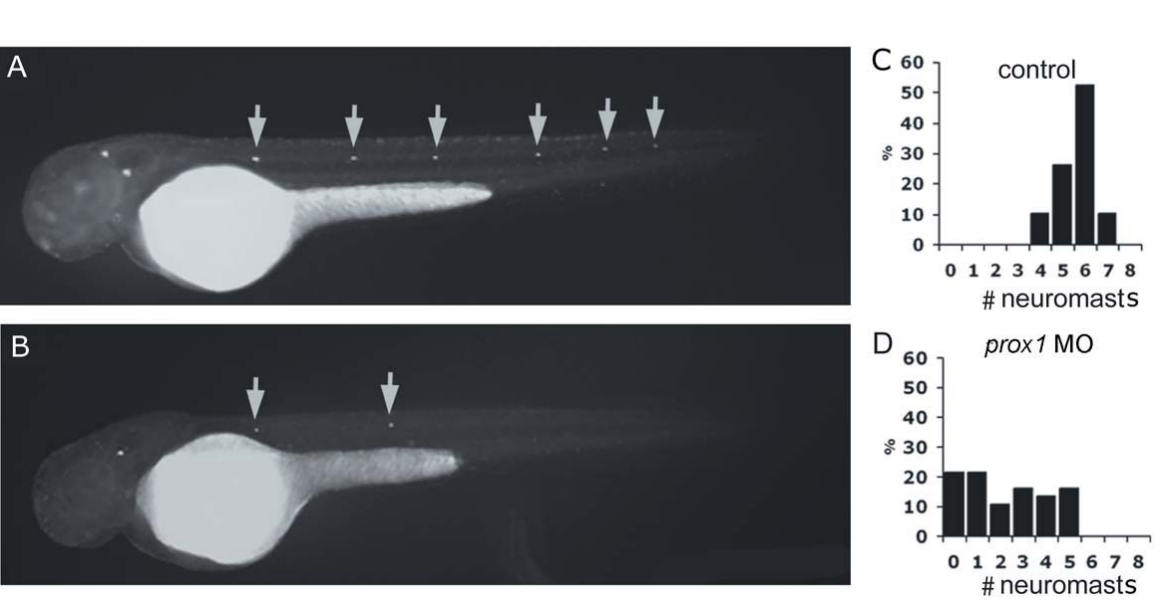
**Diasp staining in control and *prox1 *loss of function embryos at 48 hpf**. (A, B) Microinjection of *prox1 *MO decreases the number of Diasp positive cells in comparison to control embryos at the same developmental stage. (C, D) The number of Diasp-labeled neuromasts per larva at 48 hpf were counted and larvae were classified according to the number of neuromasts present on one side. While most control larvae show between 5 and 8 neuromasts (C), *prox1 *MO injected larvae display between 0 and 5 neuromasts per side (D).

Analysis of lateral line development in *prox1 *loss- and gain-of-function animals was first carried out by staining the larvae with DiAsp, a vital stain for mature and functional hair cells [6]. At 48 hpf, neuromasts of the primary lateral line system have been deposited and functional hair cells incorporate DiAsp in neuromasts of control fish. 90% of the control MO injected embryos (n = 80), presented between 5 and 8 labeled neuromasts per side at this stage (Fig. 3A; quantification in 3C). In contrast, only 15% of *prox1 *MO injected embryos showed a nearly normal number of labeled neuromasts (5), 65% presented a reduced number of labeled neuromasts (between 1 and 4 labeled neuromasts per side) and 20% a complete absence of labeling (n = 76) (Fig. 3B and 3D). The effect of *prox1 *loss of function was not due to developmental delay: at 72 hpf, MO injected embryos still presented less neuromasts than control MO injected embryos [see Additional file 2]. Gain of function experiments by means of *prox1 *mRNA injection did not significantly affect DiAsp labeling of neuromasts (data not shown).

**Figure 4:**
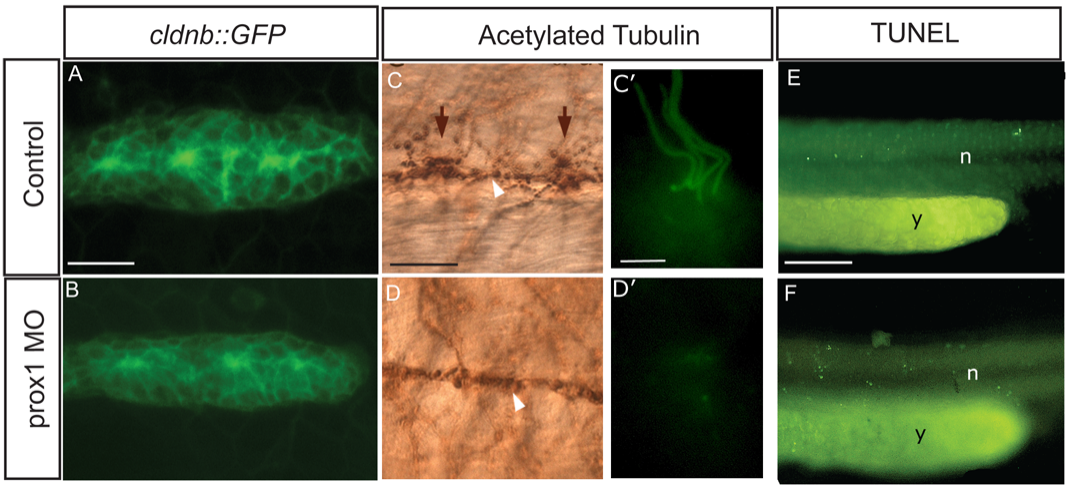
***prox1 *loss of function of does not affect PLL primordium cell number or PLL nerve development**. (A, B) GFP labeled primordia migrating at 32 hpf in *cldnb::GFP *transgenic fish. The size of the primordium is not affected in morphant embryos in comparison to control embryos at the same developmental stage. (C, D, C', D') Acetylated tubulin immunostaining indicates that the lateral line nerve is not perturbed in morphant embryos (white arrows) while differentiated hair cell with their kinocilia (brown arrows C, D and in fluorescence at higher magnification C', D'), are absent in morphant fish in comparison to control embryos. (E, F) *prox1 *MO injected embryos do not show increased cell death as indicated by the TUNEL assay, in comparison to control embryos. n, notochord; y, yolk. Scale bar = 15 micron in A, B, C, D, E, F and 3 micron in C', D'.

While our results indicated that hair cell development is impaired when *prox1 *expression is reduced, it did not clarify whether the phenotype was due to defective migration of the PLL primordium, aberrant deposition of neuromasts or a failure of hair cell differentiation within neuromasts. To discriminate between these possibilities, we took advantage of the *claudinB::GFP *transgenic line, that expresses GFP in the migrating primordium, and we injected the control and *prox1 *morpholinos in these fish. Analysis of GFP expression in both groups of animals showed that the number of cells, shape of the primordia, and neuromast deposition were indistinguishable between them (Fig. 4A, B and data not shown). We fixed these fish and carried out immunostaining with an antibody against acetylated tubulin, which labels neural processes (axons and dendrites of the PLL neurons) and the differentiated hair cells, identified by means of the label in their kinocilia [8]. The PLL nerve, which innervates the neuromasts, is intact in morphant larvae (compare Fig. 4C to 4D, white arrowheads) indicating that the PLL neurons and neural process formation are unaffected by *prox1 *loss-of-function. However, tubulin staining in differentiated hair cells was absent in morphant fish (compare Fig. 4C-C' with 4D-D', arrows). We were certain of the position of the neuromasts in these fish as the GFP label was still visible after immunostaining (not shown). Importantly, TUNEL assays (Fig. 4E, F) showed no differences in cell death levels, in the lateral line or elsewhere, between control and *prox1 *MO injected fish, indicating that the absence of hair cell kinociliae in morphants was not likely due to death of the hair cells after neuromast deposition.

**Figure 5:**
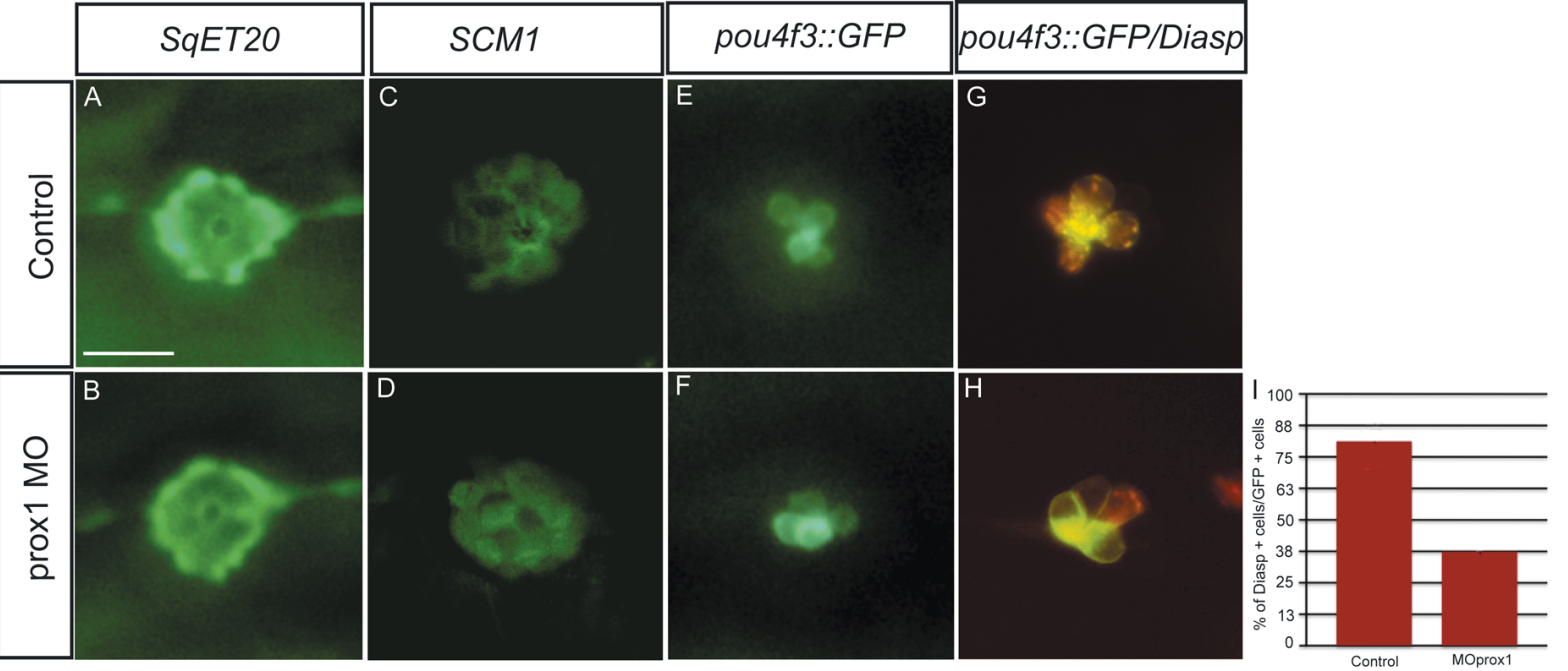
***prox1 *loss of function affects development of functional hair cells in zebrafish lateral line neuromasts**. Comparison of GFP expression between morphant and control embryos at 60 hpf shows no differences in mantle cells (A, B), progenitor cells (C, D) and hair cells (E, F) in different transgenic backgrounds, indicating that there is no effect of the *prox1 *morpholino in the specification of these cell types in the neuromast. (G, H) However, the vital stain Diasp indicates that almost 50% of the hair cells present in a neuromast are not functionally active. (I) Comparison of the percentage of GFP labeled hair cells that are co-labeled with Diasp in control and *prox1 *MO injected embryos. Scale bar = 10 micron.

To examine in more detail the *prox1 *morphant phenotype, we carried out injection of control and *prox1 *antisense morpholinos in the *SqET20 *and *SCM1 *transgenic lines. Comparison of control and morphant neuromasts in these fish at 60 hpf did not show any significant differences in number and appearance of labeled cell types (mantle cells, supporting cells and progenitors), indicating no essential role for Prox1 in their development (compare Fig. 5A to 5B and 5C to 5D). Since we had observed loss of acetylated tubulin and DiAsp staining in morphant neuromasts, we examined GFP expression in *pou4f3::mGFP *transgenic fish injected with control and *prox1 *morpholinos. Again, in this line, the number of GFP labeled cells was not significantly altered by *prox1 *loss of function (compare Fig. 5E to 5F, N = 49 embryos). The *pou4f3 *promoter-enhancer directs GFP expression to developing and mature hair cells. To distinguish fully differentiated (functional) from immature hair cells, DiAsp staining should be used. When we labeled *pou4f3::mGFP *transgenic control and morphant fish with DiAsp, a clear difference in the number of DiAsp labeled hair cells was observed between both conditions (compare Fig. 5G to 5H). While control embryos presented 75% of the *pou4f3::mGFP *positive cells also positive for DiAsp staining, in *prox1 *MO injected embryos the percentage was only 38%, indicating that these hair cells are unable to reach full functionality in the absence of Prox1 (quantification shown in Fig. 5I; n = 25 neuromasts observed for each condition). As only a fraction of *pou4f3::mGFP *positive cells were stained with DiAsp in morphant fish, we conclude that development of hair cells in morphants is arrested prior to their final differentiation and are thus unable to reach full functionality in the absence of Prox1. Our results suggest that *prox1 *has a role in the late stages of hair cell differentiation, when they acquire the mechanotransduction capacity.

## Conclusion

Overall, our studies reveal that *prox1 *mRNA and protein are expressed in the migrating PLLP and in deposited neuromasts, in particular in the progenitor/supporting cell layer and in hair cells. It is noteworthy that Prox1 protein levels and distribution were distinct from mRNA distribution suggesting that regulation of this gene at the transcriptional and posttranscriptional levels may be highly dynamic. We relied on gene inactivation and overexpression to analyze the role of *prox1 *during PLLP migration, neuromast deposition and differentiation. Interestingly, primordium migration and deposition, and differentiation of most cells types are not controlled by this gene. In other model systems, the presence and activity of Prox1 in progenitor cells directs cell fate selection: while cells with high Prox1 levels become hair cells, cells with low Prox1 levels acquire supporting cell or other fates. Interestingly, Prox1 protein localization in supporting cells may play a role in the switch from proliferation to differentiation that leads to the development of functional hair cells. In fact, in other organisms, nuclear accumulation of Prospero/Prox1 protein has been argued to regulate genes specific for the differentiated state, while in proliferating cells the protein remains in the cytoplasm [11,15,16]. In our study, *prox1 *loss-of-function causes defects in the functionality of hair cells in the neuromasts, as assayed by incorporation of DiAsp, a vital dye that is likely to enter hair cells through the mechanotransduction channel [6,42-44]. However, GFP expression driven by a regulatory element active during initial stages of hair cell differentiation is not affected by absence of Prox1. Therefore, initial hair cell specification does not appear to require *prox1*, but only terminal differentiation. It will be of interest to dissect the exact molecular players that are regulated by this gene in the zebrafish.

## Methods

### Fish and embryo maintenance

Wild type fish of the AB strain were maintained at 28°C on a 14 h light/10 h dark cycle. Embryos were collected by natural spawning, staged according to Kimmel and colleagues [45], and raised at 28°C in fish water (Instant Ocean, 0,1% Methylene Blue) in Petri dishes. Embryos used in whole-mount *in situ *hybridization were raised in 0,003% PTU (Sigma) to prevent pigmentation. We express the embryonic ages in hours post fertilization (hpf) or days post fertilization (dpf). The transgenic lines used in this study are *SqET20 *[31]; *claudinB::GFP *[34], *SCM1 *[35], *pou4f1::GFP *[32] and *pou4f3::GFP *[36]. Zebrafish (*Danio rerio*) were raised and maintained in agreement with local and national sanitary regulations.

### Whole-mount *in situ *hybridization and immunohistochemistry

Whole mount *in situ *hybridization (WISH), was carried out as described [46] on embryos fixed for 2 h in 4% paraformaldehyde/phosphate buffered saline (PBS), then rinsed with PBS-Tween, dehydrated in 100% methanol and stored at -20°C until processed for WISH [47]. Antisense riboprobes were previously *in vitro *labeled with modified nucleotides (*i.e*. digoxigenin, fluorescein, Roche). For immunohistochemistry, the following antibodies were used: rabbit anti-Prox1 (Chemicon AB5475) at a dilution of 1:250; mouse anti-GFP (Chemicon MAB3580) at a dilution of 1:200, Alexa Fluor 594 rabbit (Invitrogen A31632) at a dilution of 1:200; Alexa Fluor 488 mouse (Invitrogen A11029) at a dilution of 1:200.

### Loss- and gain-of-function analysis

For loss- and gain-of-function experiments, specific *prox1 *morpholino (*prox1 *MO) and capped RNA were injected as previously described [25]. Two *prox1 *morpholinos were designed to knockdown translation of the Prox1 protein. *prox1 *MO was directed against the translation initiation region of the mRNA (5'-ATGTGCTGTCATGGTCAGGCATCAC-'3) while *prox1 *MO splice was designed to bind to the donor splice site between exon 2 and intron 3 (5'-GGAACCTAGCCAGAAAGAAAGGACT-'3). Both were injected at a concentration of 8 ng into one-cell stage embryos.

### DiAsp

The neuromast hair cells were labeled in live embryos or larvae with 4-(4-diethylaminostyryl)-N-methylpyridinium iodide (Di-Asp; Sigma D3418, USA) as described in Collazo et al. [6]. For live staining, 48-72-hpf larvae were incubated in 5 mM Di-Asp in embryo medium for 5 min and then rinsed with fresh medium and visualized under fluorescent light in a dissection microscope. For carrying out statistical tests we counted Di-Asp-stained hair cells in the P1 neuromast (see neuromast nomenclature in Harris and collegues [48] on one side of each larva. To determine significance of differences, we used the Student's t test (SigmaStat 3.1).

## Authors' contributions

MA, EV, AP, CF and FC designed the study. CF, AP and PC carried out functional studies. CF and PC performed immunohitochemistry experiments on transgenic lines. AP, CF and MA drafted the manuscript. All authors read and approved the final manuscript.

## Supplementary Material

Additional file 1 **Decreased levels of Prox1 protein in *prox1 *loss of function embryos**. Immunohistochemistry using an anti-Prox1 antibody at 48 hpf (A) Prox1 protein distribution in control embryos in comparison to *prox1 *MO injected embryos (B) black arrow lens (l); arrowhead diencephalon (d), brown arrow diencephalic-mesencephalic boundary (dmb). Scale bar = 200 micron Click here for file

Additional file 2 **DiAsp staining in control and *prox1 *loss of function embryos at 72 hpf**. As at 48 hpf, also at 60 and 72 hpf, *prox1 *MO injected embryos still presented a decrease number of DiAsp positive cells in neuromasts in comparison to control embryos at the same developmental stage, indicating that the effect is not due to developmental delay of morphant embryos. Click here for file
