# Supplementary figures and images for: The zebrafish prospero homolog prox1 is required for mechanosensory hair cell differentiation and functionality in the lateral line
Source: BMC Dev Biol. 2009 Nov 30;9:58. doi: 10.1186/1471-213X-9-58 (PMC2794270; doi:10.1186/1471-213X-9-58)

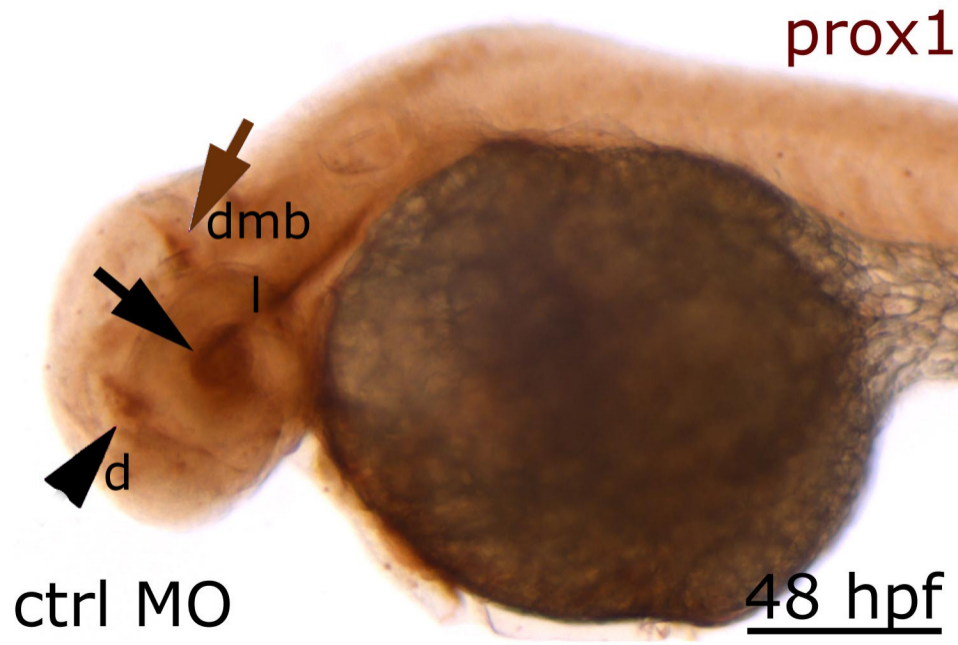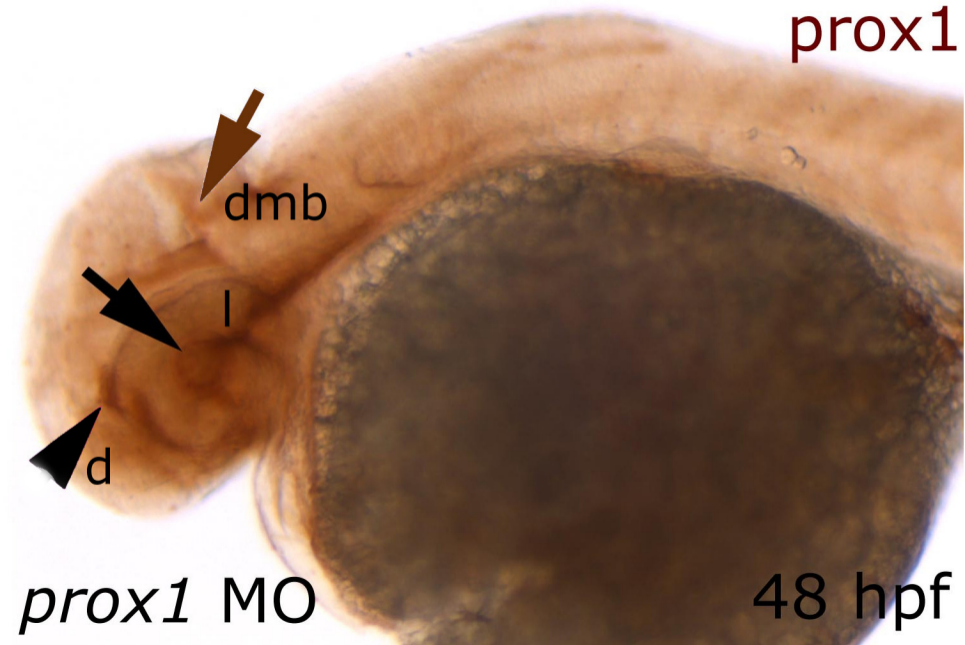

Supplement: Additional file 1 — Decreased levels of Prox1 protein in prox1 loss of function embryos. Immunohistochemistry using an anti-Prox1 antibody at 48 hpf (A) Prox1 protein distribution in control embryos in comparison to prox1 MO injected embryos (B) black arrow lens (l); arrowhead diencephalon (d), brown arrow diencephalic-mesencephalic boundary (dmb). Scale bar = 200 micron [file 1471-213X-9-58-S1.PDF]

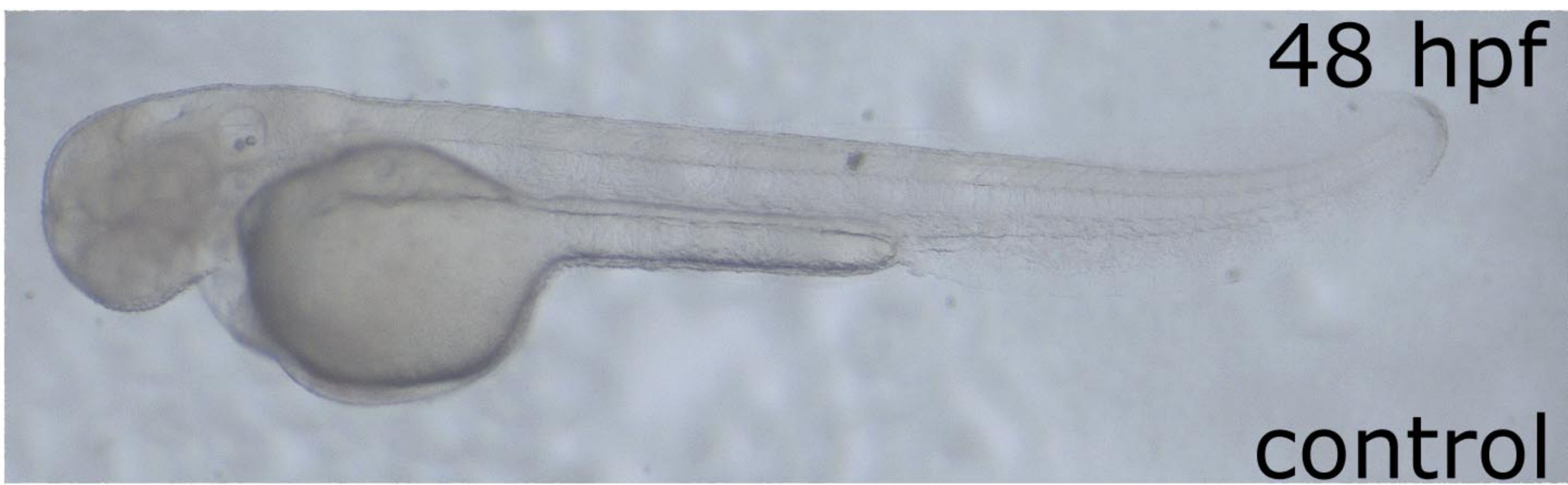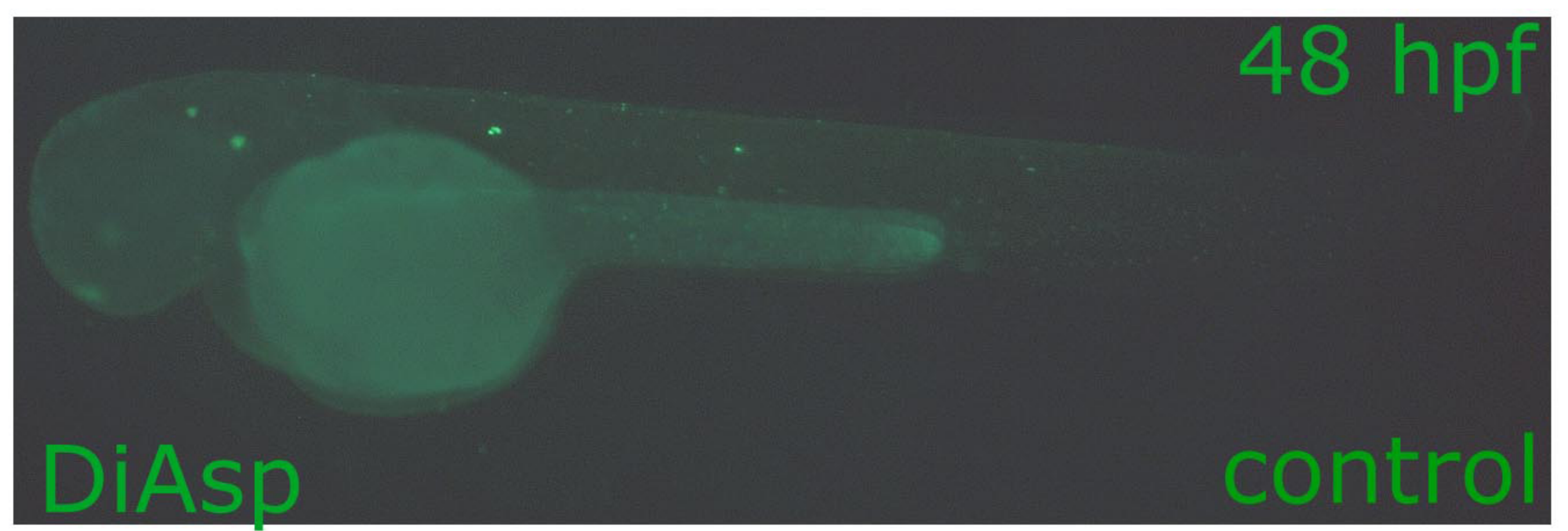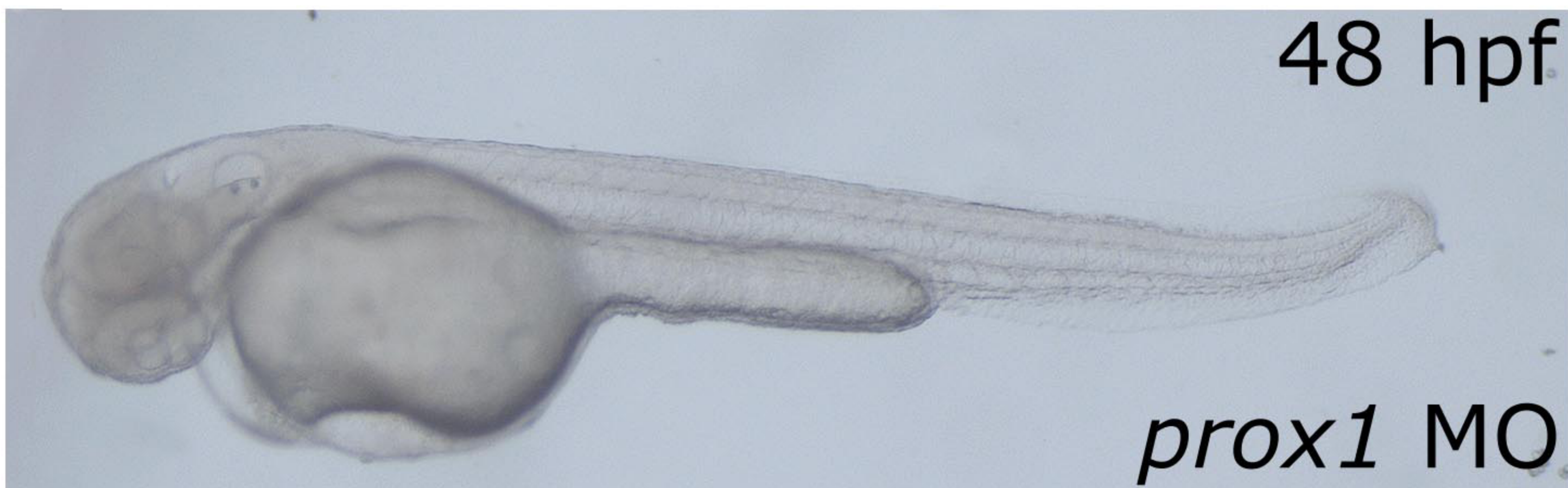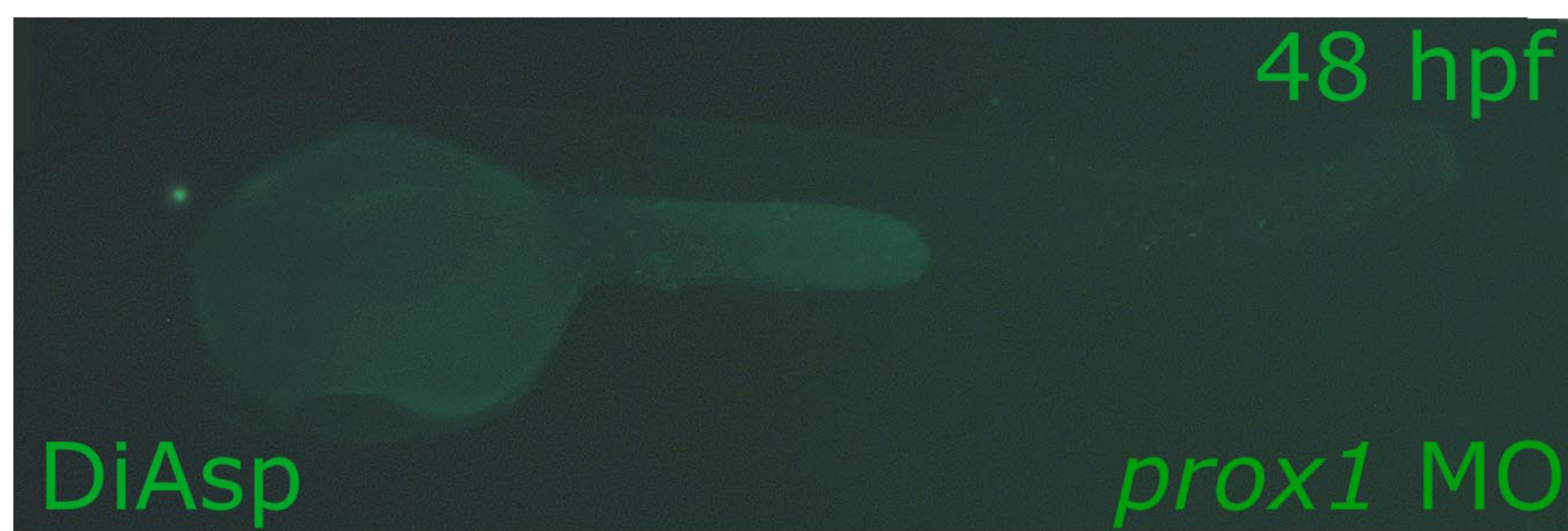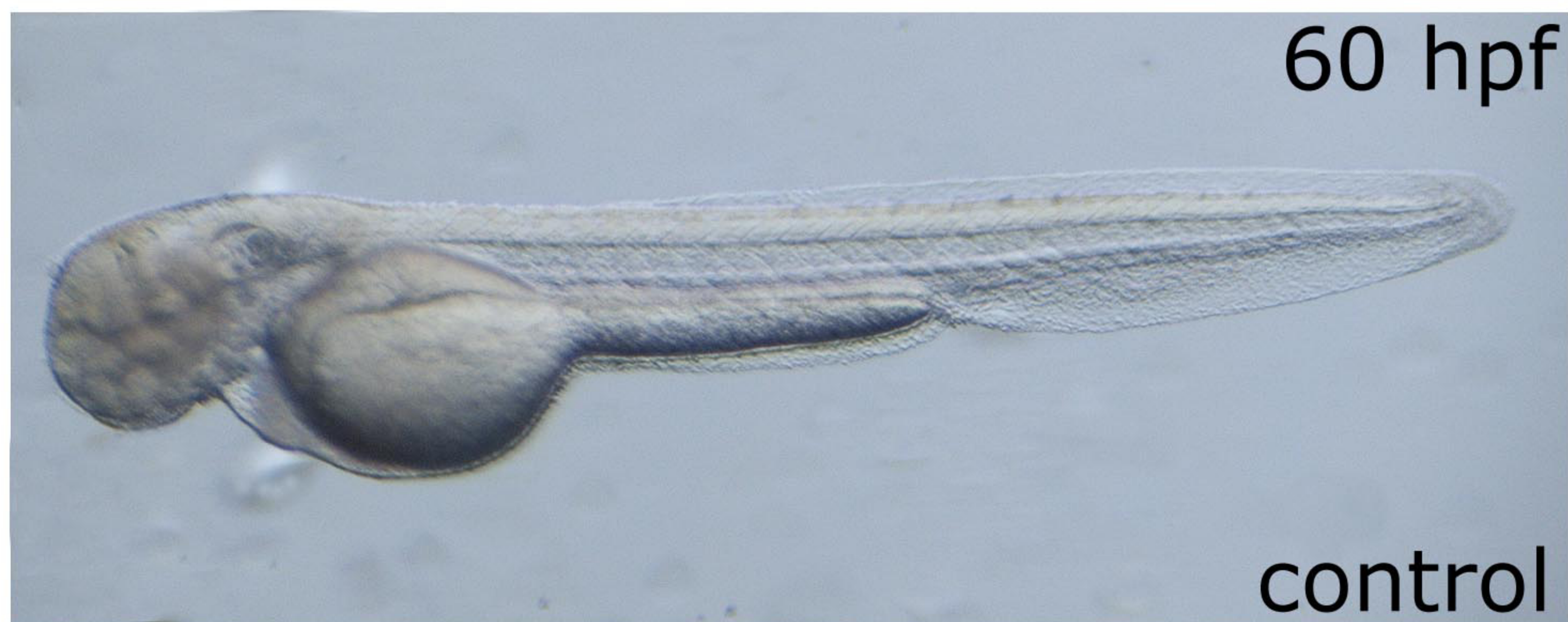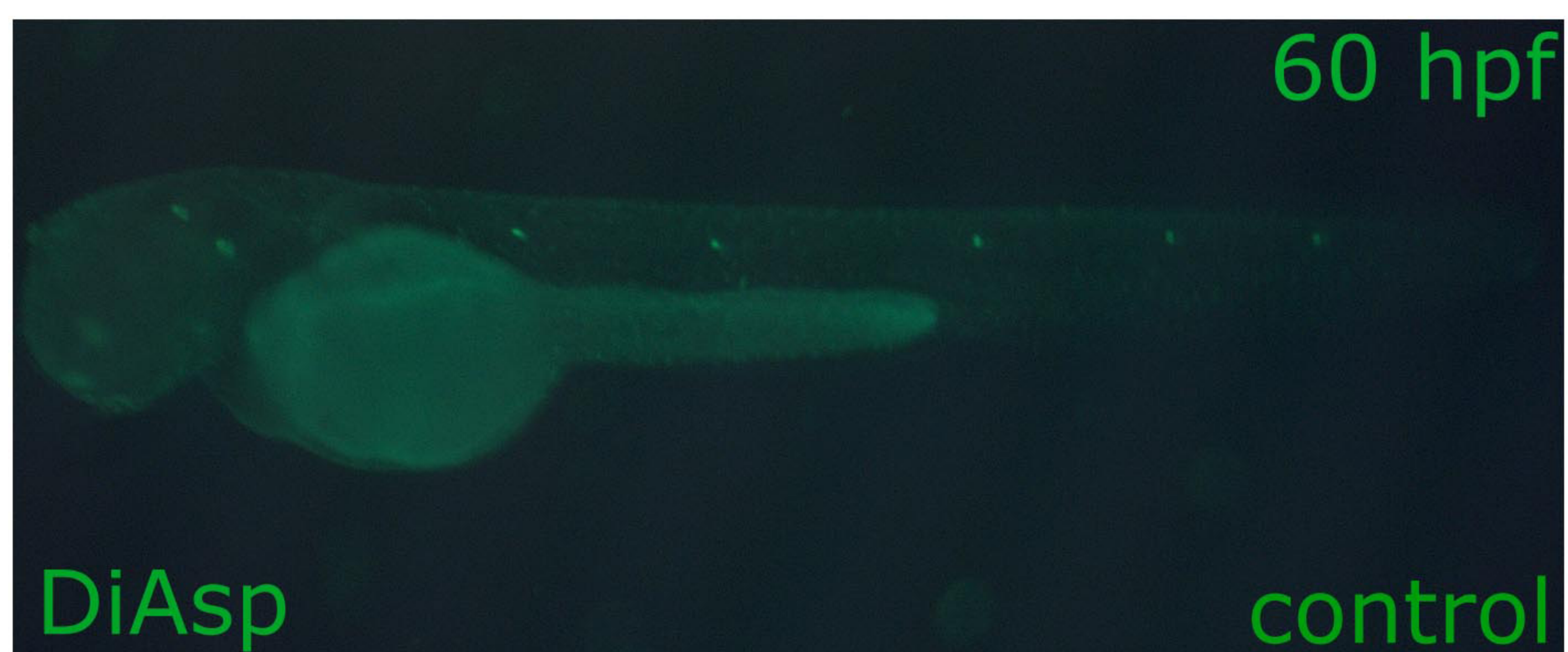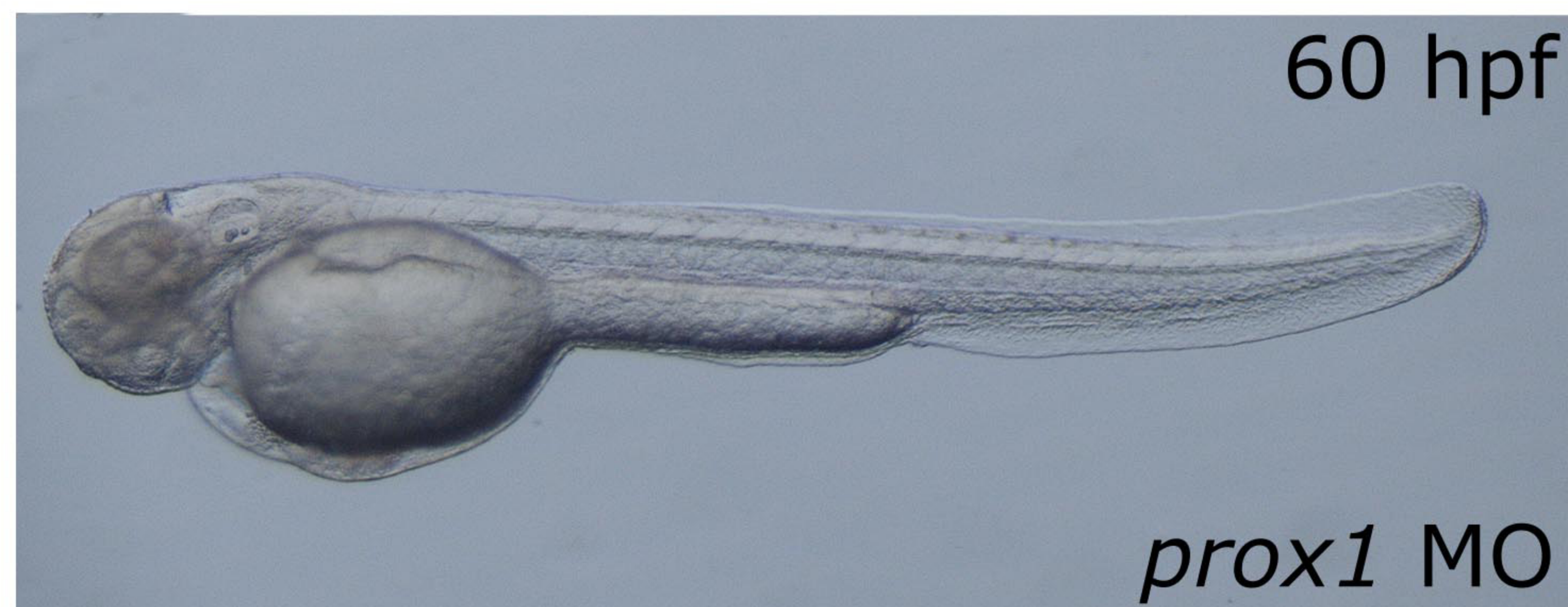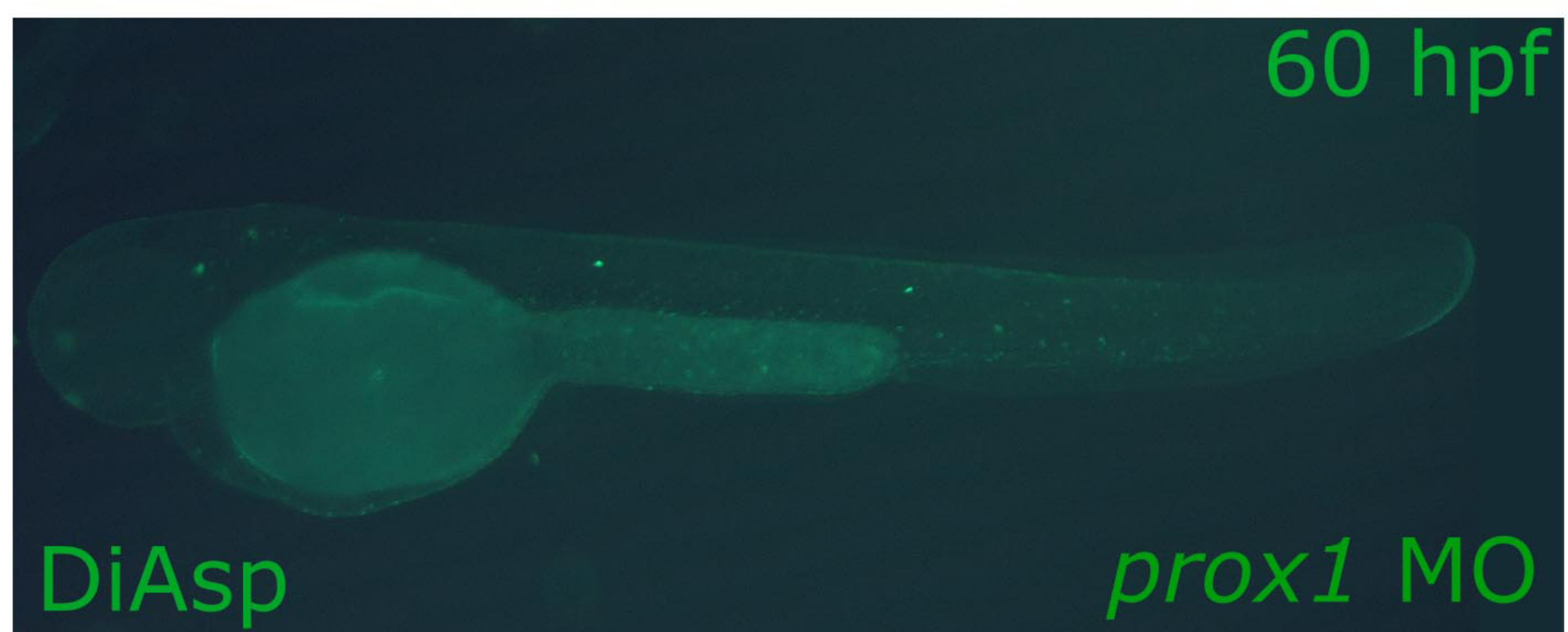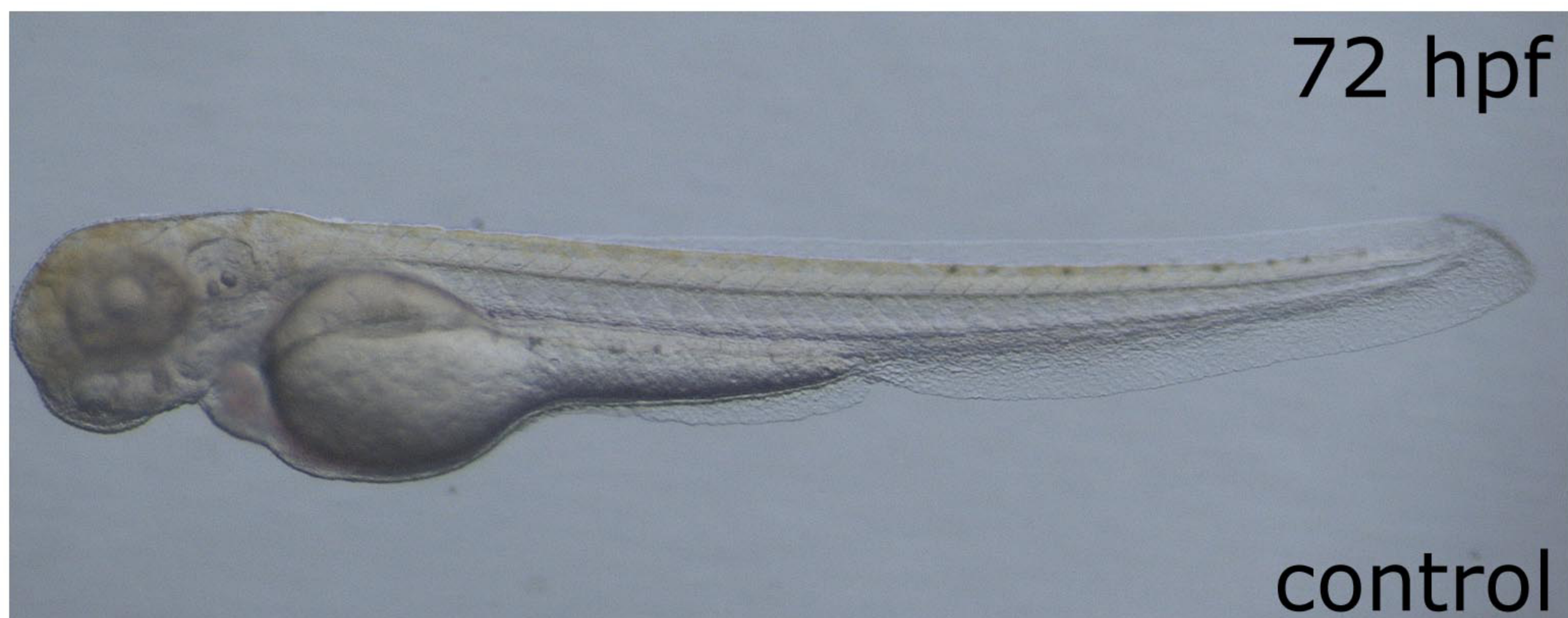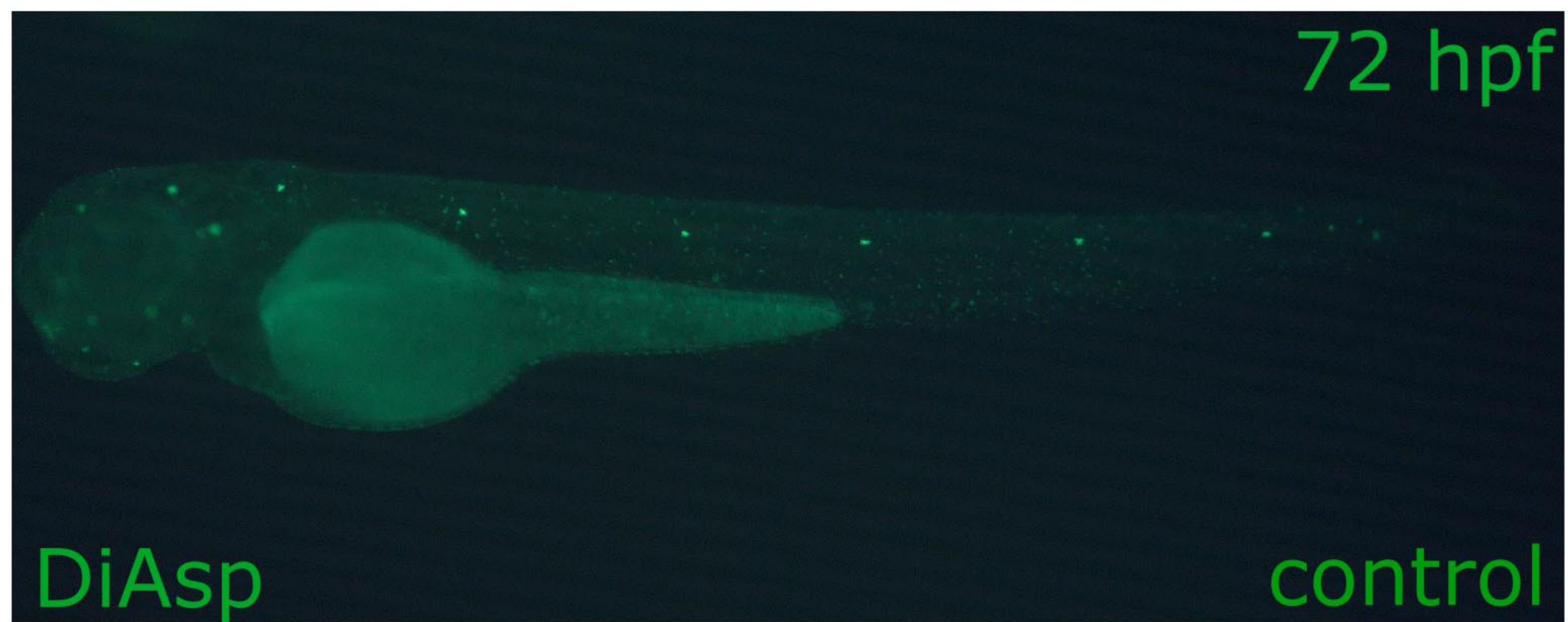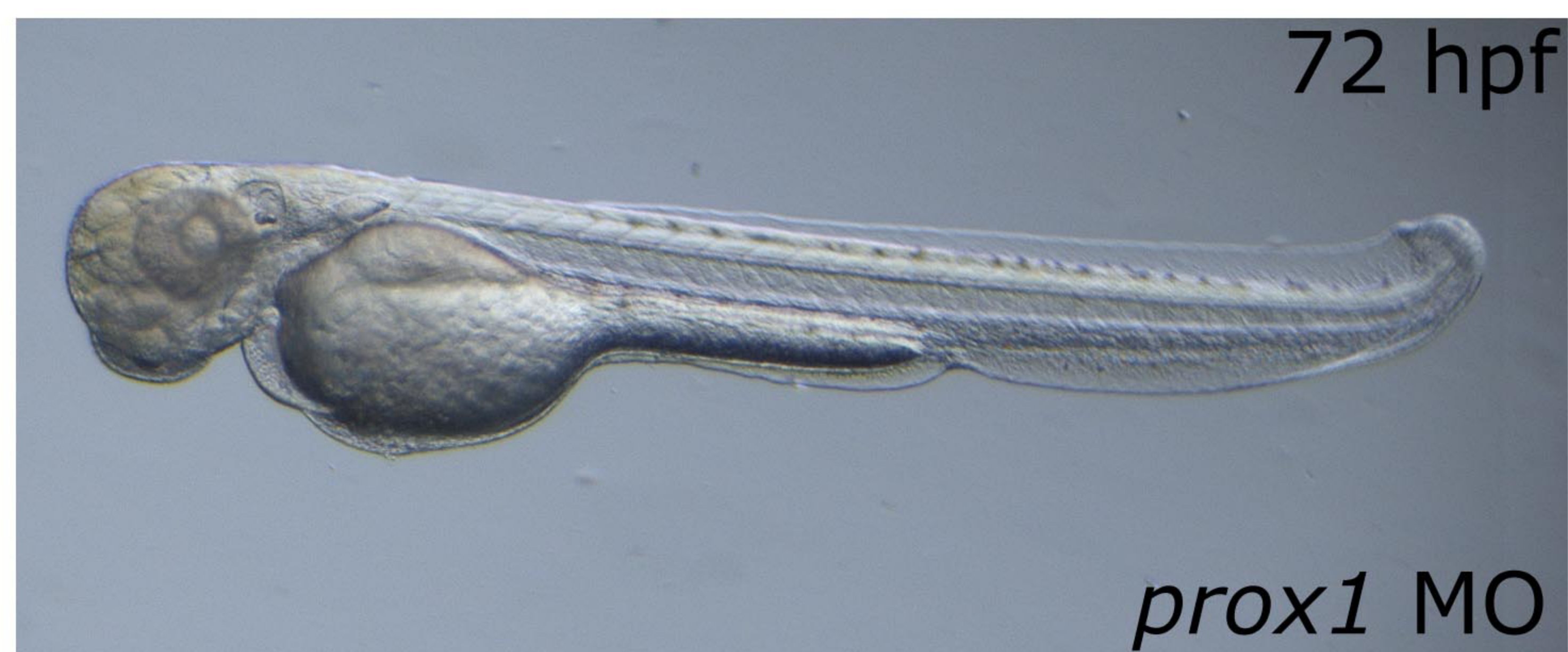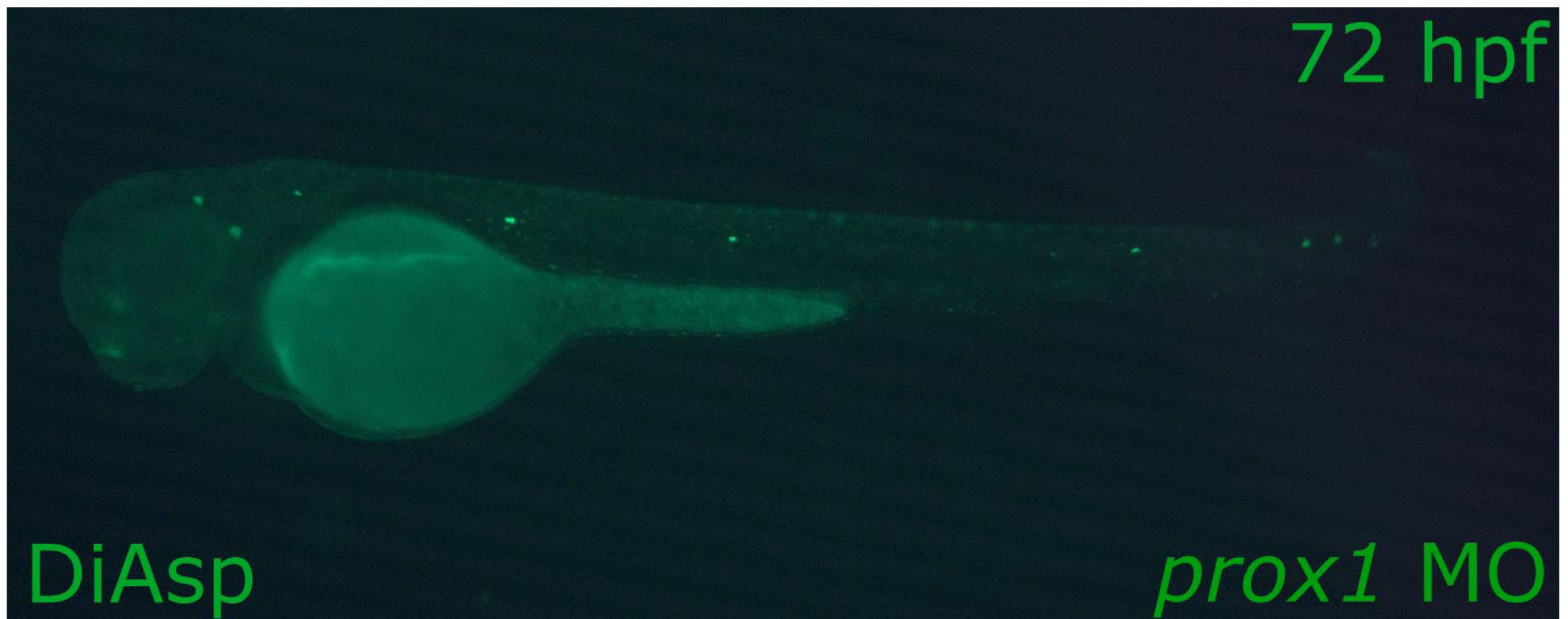

Supplement: Additional file 2 — DiAsp staining in control and prox1 loss of function embryos at 72 hpf. As at 48 hpf, also at 60 and 72 hpf, prox1 MO injected embryos still presented a decrease number of DiAsp positive cells in neuromasts in comparison to control embryos at the same developmental stage, indicating that the effect is not due to developmental delay of morphant embryos. [file 1471-213X-9-58-S2.PDF]
